# A Strategy for Screening and Confirmation of HTLV-1/2 Infections in Low-Endemic Areas

**DOI:** 10.3389/fmicb.2020.01151

**Published:** 2020-06-03

**Authors:** Huimin Ji, Le Chang, Ying Yan, Xinyi Jiang, Huizhen Sun, Fei Guo, Lunan Wang

**Affiliations:** ^1^National Center for Clinical Laboratories, Beijing Hospital, National Center of Gerontology, Institute of Geriatric Medicine, Chinese Academy of Medical Sciences, Beijing, China; ^2^Beijing Engineering Research Center of Laboratory Medicine, Beijing Hospital, Beijing, China; ^3^Graduate School, Peking Union Medical College, Chinese Academy of Medical Sciences, Beijing, China

**Keywords:** T-cell lymphotropic virus type 1/2 (HTLV-1/2), enzyme-linked immunosorbent assay (ELISA), chemiluminescence immunoassay (CLIA), electrochemiluminescence immunoassay (ECLIA), western blot, line immunoassay

## Abstract

Serological tests have been widely used for detecting human T-cell lymphotropic virus type 1/2 (HTLV-1/2) antibodies in the endemic areas, but their performance in low-risk populations is rarely reported. The aim of this study was to evaluate the performance of four HTLV-1/2 screening assays and to discuss a strategy for diagnosis of HTLV-1/2 infection in a non-endemic area. At the present study, 1546 specimens repeatedly reactive (RR) by one screening ELISA were collected from blood centers/banks from January 2016 to April 2019. Avioq-ELISA, Murex-ELISA, Roche-ECLIA and Fujirebio-CLIA were independently performed on each plasma sample and compared to WB and LIA confirmatory tests. Positive or indeterminate specimens with blood available were quantified by qPCR. The results showed that 48 samples were finally confirmed as HTLV-1 positive, 13 were HTLV positive, 151 were indeterminate, and 387 were negative. All the WB-positive samples were also LIA-positive. Roche-ECLIA showed the highest sensitivity that was able to detect 91.8% positives and combined with the Murex-ELISA would significantly increase the positive detection rate (98.4%). In addition, LIA yield more indeterminate and HTLV-untyped results than WB (152 vs. 27), but was able to resolve infection status of some individuals with an indeterminate WB. Besides, 3 WB indeterminate and 1 LIA-untyped samples were confirmed as HTLV-1 positive by qPCR. Based on these findings, we put forward a proper test strategy for HTLV-1/2 diagnosis in low-prevalence areas. If possible, the Roche-ECLIA with the highest sensitivity is suggested as a second screening assay in primary labs. If not, all RR specimens are recommended to be firstly retested by Roche-ECLIA and Murex-ELISA in the reference lab. Secondly, samples reactive to any one of the two tests were quantified by qPCR, and then the NAT-negatives were furtherly submitted to LIA for confirmation. Thereby, the cost can be reduced and the diagnostic accuracy would be improved.

## Introduction

Human T-lymphotropic virus (HTLV) was the first RNA retrovirus to be associated with cancer (Poiesz et al., 1980). To date, four HTLV related viruses (types 1 to 4) have been discovered, but only HTLV-1 and HTLV-2 have been convincingly linked to human diseases (Poiesz et al., 1980; Kalyanaraman et al., 1982; Calattini et al., 2005; Wolfe et al., 2005). HTLV-1 is the etiologic agent of Adult T-cell leukemia (ATL) and HTLV-associated myelopathy/tropical spastic paraparesis (HAM/TSP) (Verdonck et al., 2007). HTLV is mainly spread via mother-to-child transmission, sexual contact, and through contaminated needles shared by drug users. It also can be transmitted through the transfusion of infected blood components and tainted liver, kidney, or lung transplants. To mitigate these risks, mandatory screening of blood supplies for HTLV-1/2 was implemented in the mid-1980s in most developed and several developing countries (Williams et al., 1988; Maeda, 1989). However, because most regions of China have long been considered non-endemic for HTLV, limited screening has been implemented.

Currently, initial diagnosis of HTLV-1/2 infection is mainly based on detecting specific antibodies in plasma or serum using enzyme-linked immunosorbent assay (ELISA) and CLIA or ECLIA. Several commercial kits based on recombinant and/or synthetic peptide antigens alone or in combination with viral lysates have also been adapted for large-scale screening of HTLV-1/2 antibodies. However, the kits fail to differentiate between HTLV-1 and HTLV-2 infections because the two types share a high homology between them (Andersson et al., 1999; da Silva Brito et al., 2018). Besides, the high false-positive rate of these commercial assays, especially in low-seroprevalence populations, is a major problem. Therefore, confirmatory assays with high specificity are required for samples exhibiting a signal repeatedly greater than or equal to the threshold value of the screening assays.

Western blot is most frequently used to confirm the presence of anti-HTLV-1/-2 antibodies. The FDA-licensed MP Diagnostics HTLV Blot 2.4 uses a combination of recombinant HTLV-1/2 proteins and HTLV-1 viral lysate to improve sensitivity. This assay also uses HTLV type-specific recombinant envelope protein (gp46-1 and gp46-2) to discriminate viral types (Varma et al., 1995). However, a large number of WB-indeterminate and WB-untypable results are commonly found in several population groups and especially among low-risk blood donors (Mahieux et al., 2000; Ando et al., 2004; Yao et al., 2006). To address this shortcoming, another serologic confirmatory test with improved sensitivity, INNO-LIA HTLV, has been developed to confirm and differentiate HTLV-1 and HTLV-2 infections (Zrein et al., 1998). This assay appeared useful in reducing the numbers of inconclusive WB results (Zrein et al., 1998).

Molecular assay is another method used for HTLV confirmation. Real-time PCR or quantitative PCR (qPCR) have been used to determine the real status of HTLV infections and quantify the proviral load (PVL). However, these molecular assays have had low sensitivity in special population groups, such as individuals infected with HIV or HTLV-2 (Montanheiro et al., 2008; Campos et al., 2017).

Although several commercially available HTLV-1/2 diagnostic assays have been used worldwide, their performance in endemic and non-endemic areas varies greatly. Moreover, few studies have been conducted to compare the performance of LIA and WB in low-prevalence populations. Therefore, the objective of this study was to evaluate several FDA- or CE-licensed HTLV-1/2 assays to diagnose the specimens that were RR to one ELISA, and to discuss strategies for the diagnosis of HTLV-1/2 infection in a non-endemic area.

## Materials and Methods

### Samples and Study Design

**FIGURE 1:**
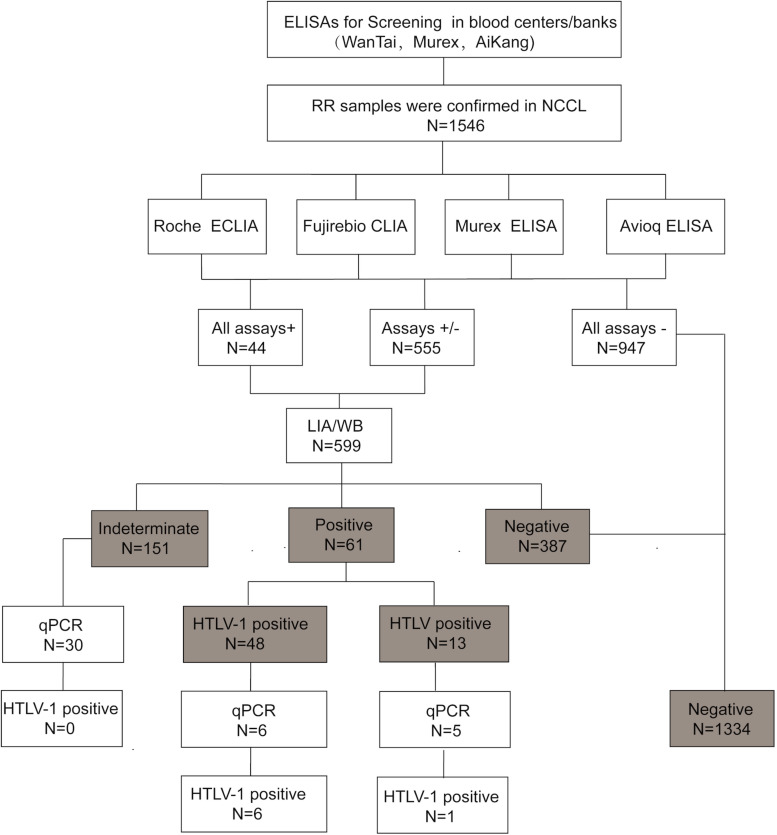
The testing algorithms of HTLV-1/2 in our study. WB, western blot; ELISA, enzyme-linked immunosorbent assays; CLIA, chemiluminescence immunoassays; ECLIA, electrochemiluminescence immunoassays; RR, repeatedly reactive. +: positive/reactive; –: negative/non-reactive; +/–: indeterminate.

All specimens were collected from eligible donors from blood banks/centers in 26 provinces in China between January 2016 and April 2019. The RR samples were delivered at 2–8°C or frozen to the NCCL for confirmation. The RR samples were also HBsAg, anti-HCV, anti-HIV, and anti-TP negative by both two screening ELISAs in the enrolled blood centers. According to the confirmatory process (Figure 1), all RR samples were simultaneously subjected to four screening assays in the NCCL. Samples reactive to any one of the four assays were further confirmed by a LIA (INNO-LIA HTLV I/II score, Fujirebio, Japan) and WB (MP HTLV Blot 2.4, MP Biomedicals, Singapore).

Samples with limited plasma volumes that were not enough for all assays were excluded from our study. Enrolled samples with a positive result either by WB or LIA were classified as positive. Positive or indeterminate specimens with whole blood available were further quantified by qPCR in our lab. In addition, blood donations with an indeterminate result were recollected from the same donor, and retested by the same detection process.

### Screening and Evaluated Assays

Three different kinds of commercially available ELISA assays [Wantai HTLV-1/2 antibody ELISA kits (Wantai BioPharm, China), Murex HTLV I+II (Diasorin S.p.A., United Kingdom) and Foresight HTLV-1/2 antibody ELISA kits (Acon Biotech, China)] were used for screening HTLV1/2 antibodies at all participating blood centers/banks in China. Reactive specimens were retested by the same assay and any one of two-round retests found to be reactive were defined as RR specimen.

Further detection was done in the NCCL using Elecsys HTLV-I/II (Roche Diagnostics, Germany), Lumipulse G HTLV-I/II (Fujirebio, Japan) and two kinds of ELISA assays (Murex HTLV I+II (Diasorin S.p.A., United Kingdom) and Avioq HTLV-I/II Microelisa System (Avioq, Durham, NC, United State). The key features of the evaluated assays are summarized in Table 1.

**TABLE 1:** Technical features of evaluated assays and confirmatory assays used in the study.

Assay name	Manufacturer	Assay type	Detection antigens	Testing time
Elecsys HTLV-I/II	Roche diagnostics	One-step double-antigen sandwich chemiluminescent immunoassay	Viral recombinant antigens gp21 and p24	18 min
Lumipulse G HTLV-I/II	Fujirebio	Two-step sandwich chemiluminescent immunoassay	Recombinant antigens p19 I/II, p24 I/II, gp46 I/II, gp21 I	4 min
Avioq HTLV-I/II Microelisa System	Avioq	ELISA	Viral antigens (purified viral lysate) and recombinant HTLV-1 p21E antigen	150 min
Murex HTLV-I/II	Diasorin	ELISA	HTLV-1 and HTLV-2 antigens	90 min
INNO-LIA HTLV I/II score	Fujirebio	A line immunoassay	Recombinant antigens p19 I/II, p24 I/II, gp46 I/II, gp21 I/II	18 h
MP HTLV Blot 2.4	MP Biomedicals	Western Blot assay	Recombinant HTLV-1/2 antigens and HTLV-1 viral lysate	150 min

*ELISA, enzyme-linked immunosorbent assay.*

### Confirmatory Tests

In the LIA, the strips contained recombinant proteins (rp19 I/II, rp24 I/II, and rgp21 I/II) for confirmation and synthetic peptides (gp46 I, p19 I, and gp46 II) for differentiation of HTLV-1 and HTLV-2 antibodies. Samples were considered HTLV positive if they were reactive with at least two confirmation bands, including rgp21 I/II. The positive samples were typed as HTLV-1 if the intensities of gp46 I and p19 I bands were equivalent to or higher than that of the gp46 II band. HTLV-2 positivity was defined if the gp46 II band were more intense than the gp46 I and p19 I bands and if no discriminate bands appeared, an untypable HTLV result was denoted. A specimen was classified as indeterminate if it were reactive with one band (rgp21 I/II) or two bands (except rgp21 I/II). Samples were considered negative if a single rp24 I/II, rp19 I/II, gp46 I, or gp46 II band or no bands appeared.

WB is most frequently used as a supplemental assay for confirming and differentiating the presence of antibodies against HTLV. Results of MP HTLV Blot 2.4 were interpreted according to the stringent criteria provided by the manufacturer. Briefly, HTLV-1 seropositivity was defined as reactivity to gag (p19 with or without p24) and two env (GD21 and rgp46-I). HTLV-2 seropositivity was defined as reactivity to gag (p24 with or without p19) and two env (GD21 and rgp46-II). Samples that were reactive to both gag (p19 and p24) and env (GD21) were defined as HTLV seropositive but were considered untypable. Any other patterns of specific bands that did not meet the above criteria were considered as indeterminate. Absence of bands or appearance of non-specific band patterns were interpreted as negative.

### Quantitative PCR (qPCR)

DNA was extracted from whole blood of each specimen using the Tiangen Magnetic Blood Genomic DNA Kit (Tiangen Biotech, China). HTLV-1 proviral DNA was detected and quantified by qPCR using a set of primer pairs and a TaqMan probe targeting the HTLV-1 pol region. The RPPH1 gene was also amplified simultaneously as an internal control. The primers and probes are shown in Supplementary Table S1. Standard curves were generated using recombinant plasmid DNA with HTLV-1 pol and RPPH1 sequences. Normalized HTLV-1 PVL was calculated using the formula: (HTLV-1 DNA average copy number/RPPH1 average copy number) × 2 × 100 leukocytes and expressed as the number of HTLV-1 copies per 100 PBMCs. The detection limit for the HTLV-1 pol region was estimated to be 2.5 copies per 10,000 host leukocytes with a 95% hit-rate.

### Statistical Analysis

The PPVs were calculated by SPSS version 21.0 (IBM, Corp., Armonk, NY, United States). Descriptive statistics are presented as geometric means + standard deviations. Results were analyzed with *t*-test. *p* < 0.05 was considered statistically significant.

## Results

### HTLV Confirmation and Typing

A total of 1546 RR samples with enough volumes were included in the final sample count in our study. Of the 1546 samples, 555 showed discordant results and 991 showed consistent results across the four assays. Of the 991 consistent samples, 44 were reactive to all four assays while the rest were negative in all assays. Finally, 599 samples that were reactive to at least one assay were confirmed by LIA and WB. Of these samples, 73.29% (439/599) showed consistent results in the two confirmatory tests, including 44 HTLV-1 positive samples, 8 indeterminate samples and 387 negative samples (Table 2). As samples were defined as positive if any confirmatory test were positive, 48 samples were finally identified as HTLV-1 positive, 13 as HTLV positive, 151 as indeterminate and 387 as negative. In addition, 41 blood samples were also tested by qPCR, which showed that 6 HTLV-1 positive samples and 1 HTLV-untyped samples were NAT-positive and 30 indeterminate samples were NAT-negative. The results and validation algorithm are shown in Figure 1.

**TABLE 2:** INNO-LIA results compared to WB results.

WB results	LIA results				Total
	HTLV-1	HTLV	Ind	Neg	
HTLV-1	44	1	0	0	45
Ind	2	4	8	13	27
Neg	1	9	130	387	527
Total	47	14	138	400	599

*LIA, line immunoassay; WB, western blot; Ind, indeterminate, Neg, negative.*

As shown in the Table 2, 61 samples were interpreted as positive using the LIA as a reference. The positive detection rates for the four screening assays ranged from 72.1 to 91.8% (Table 3 and Supplementary Table S2). A combination of any two of the four assays increased the positive detection rate. We found that the combination of Roche-ECLIA and Murex-ELISA detected 98.4% positives, which was higher than other combinations. However, only 44 samples were classified as positive when WB was used as a standard and the positive detection rates were increased to 93.2, 97.2, 100, and 100% for Avioq-ELISA, Murex-ELISA, Roche-ECLIA, and Fujirebio-CLIA, respectively (Supplementary Table S2).

**TABLE 3:** Details of the discrepant results by LIA and WB among the finally confirmed HTLV positive samples.

Sample no.	ELISA		CLIA		LIA		WB 2.4		qPCR	
	Avioq	Murex	Roche	Fujirebio	Bands pattern	Results	Bands pattern	Results	PVL	Results
	S/CO	S/CO	S/CO	COI						
1626	1.787	3.315	427.9	50.0	p19 I/II, p24 I/II, gp46 I/II, gp21 I/II, gp46-I	HTLV-1	p19, p24, p26, p28, p32, p36, p53, pg46	Ind	0.339	HTLV-1
1705	1.166	3.297	77.8	50.0	p19 I/II, gp46 I/II, gp21 I/II, gp46-I	HTLV-1	p19, rgp46-I	Ind	11.397	HTLV-1
359	5.323	9.409	343.9	50.0	p19 I/II, p24 I/II, gp46 I/II, gp21 I/II	HTLV	p19, p24, p26, p28, p32, p36, p53, GD21, rgp46-I	HTLV-1	/	NT
1739	1.146	3.49	178.2	50.0	p19 I/II,gp21 I/II, gp46-I	HTLV	p19, p26, p28, GD21	Ind	0.009	HTLV-1
525	0.14	0.43	1.12	3.1	gp46 I/II, gp21 I/II, gp46-I	HTLV-1	/	Neg	/	NT
495	0.19	0.40	1.9	8.4	p19 I/II, gp21 I/II	HTLV	/	Neg	/	NT
1441	0.34	0.55	11.95	5.6	p19 I/II, gp21 I/II	HTLV	p19	Ind	/	Neg
1747	0.074	2.02	42.31	0.6	p19 I/II, gp21 I/II	HTLV	rgp46-II	Ind	/	Neg
53	0.142	2.212	0.215	1.8	p24 I/II, gp21 I/II	HTLV	/	Neg	/	NT
1412	0.17	2.59	0.472	1.3	p19 I/II, gp21 I/II	HTLV	/	Neg	/	NT
816	0.24	1.86	0.136	0.3	p19 I/II, gp21 I/II	HTLV	p19, p26	Ind	/	NT
1295	0.15	1.02	0.4	0.9	p19 I/II, p24 I/II, gp21 I/II	HTLV	/	Neg	/	Neg
489	0.16	0.35	5.18	0.1	p19 I/II, gp21 I/II	HTLV	/	Neg	/	NT
647	0.13	0.45	4.15	0.9	p19 I/II, gp21 I/II	HTLV	/	Neg	/	NT
1285	0.29	0.36	1.32	0.7	p24 I/II, gp21 I/II	HTLV	/	Neg	/	NT
1461	0.32	0.38	1.47	0.4	p24 I/II, gp21 I/II	HTLV	/	Neg	/	Neg
457	0.190	0.780	0.727	3.1	p19 I/II, gp21 I/II	HTLV	/	Neg	/	NT

### Correlation Between the PPVs and the Reactivity Index of the Evaluated Commercial Assays

**FIGURE 2:**
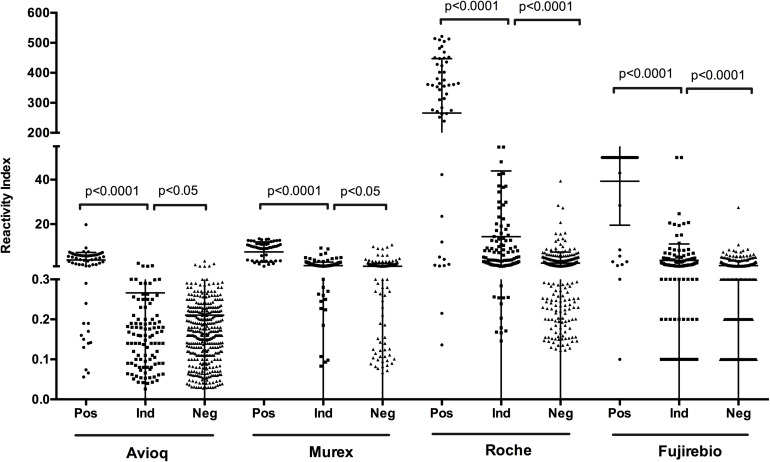
S/CO or COI values distribution among the finally confirmed positive, indeterminate and negative samples. Ind, indeterminate, Pos, positive; Neg, negative.

In each assay, positive samples confirmed by LIA and WB displayed a significantly higher reactivity index than indeterminate (*p* < 0.0001) and negative samples (*p* < 0.0001) (Figure 2). Moreover, the difference in reactivity indexes between indeterminate and negative samples as detected by the evaluated assays was also statistically significant (*p* < 0.05), indicating that reactivity indexes may correlate with the confirmatory results.

To determine the relationship between PPVs and reactivity indexes on four evaluated assays, we analyzed the results of the assays at different cut-off reactivity indexes for the four assays (Table 4). We found that when the cut-off values were 1.0, the PPV for Avioq-ELISA was 91.7%, but for Murex-ELISA, Roche-ECLIA and Fujirebio-CLIA the PPVs were only 29.5, 21.0, and 23.7%, respectively. PPVs above 95% were observed when cut-off values were 1.5 for Avioq-ELISA, 10.0 for Murex-ELISA, 29.0 for Roche-ECLIA and 8.8 for Fujirebio-CLIA. PPVs were 100% when the cut-off ratios for Avioq-ELISA, Murex-ELISA, Roche-ECLIA and Fujirebio-CLIA were 2.5, 11.0, 67.2, and 28.0, respectively.

**TABLE 4:** Relationship between S/CO or COI values and PPV.

Avioq-ELISA		Murex-ELISA		Roche-ECLIA		Fujirebio-CLIA	
S/CO	PPV	S/CO	PPV	S/CO	PPV	COI	PPV
1.00	91.7%	1.0	29.5%	1.0	21.0%	1.0	23.7%
1.05	93.6%	4.0	79.6%	20.0	90.6%	8.0	89.1%
**1**.**50**	**95.5%**	8.0	88.1%	28.0	94.1%	8.4	92.5%
1.80	97.5%	9.8	92.9%	**29.0**	**96.0%**	**8.8**	**96.0%**
2.00	97.4%	**10.0**	**96.0%**	40.0	98.0%	12.0	98.0%
**2**.**50**	**100.0%**	**11.0**	**100.0%**	**67.2**	**100.0%**	**28.0**	**100.0%**

*LIA, line immunoassay; PPV, positive predictive value. The cut-off values were showed in bold when the PPVs reached to 95% or 100%.*

### Discrepancies Between INNO-LIA and WB in the Finally Confirmed Positive Samples

Seventeen samples that were finally defined as HTLV-1 or HTLV positive showed discrepant results between INNO-LIA and WB (Tables 2, 3). Of these 17 samples, 3 were LIA HTLV-1 positive and 14 were LIA HTLV positive but untypable. Two out of the 3 LIA HTLV-1 positives were also NAT-positive but were WB-indeterminate while the other one was WB-negative. One out of the 14 LIA-untyped samples was discriminated as HTLV-1 by WB, 4 were confirmed as WB-indeterminate and 9 were WB-negative. It is noteworthy that 1 LIA-untypable but WB-indeterminate sample was also NAT-positive.

After combining the results confirmed by LIA, WB and qPCR, 5 samples were classified as HTLV-1 positive and 12 samples were HTLV positive but untypable (Table 3). The average S/CO or COI values of the untyped HTLV infections for Murex-ELISA, Roche-ECLIA and Fujirebio-CLIA were 1.03, 5.49, and 2.09, respectively. All these untyped samples did not respond to Avioq-ELISA and their positive rates for Roche-ECLIA, Murex-ELISA and Fujirebio-CLIA were 7/12, 5/12 and 5/12, respectively, indicating that these specimens may easily be missed by some screening assays.

### Characterization of Indeterminate Results

Of the 151 samples which were finally interpreted as indeterminate, 130 were LIA ± / WB-, 15 were LIA-/WB ± and 6 were indeterminate by both tests (Tables 2, 5). Most of the LIA-indeterminate samples showed a gp21 I/II pattern while only a few displayed a p19 I/II plus p24 I/II pattern. Thirty LIA-indeterminate samples were available for qPCR detection, but none were positive. In the WB-indeterminate samples, a single rgp46-II band was the most frequently observed pattern, followed by a single GD21 and a single p19 band. Of these indeterminate samples, 68.7% were Roche-ECLIA positive, 54.3% were Fujirebio-CLIA positive, 43.7% were Murex-ELISA positive and only 0.4% were Avioq-ELISA positive.

**TABLE 5:** The reactivity patterns of the indeterminate samples.

Ind. pattern	INNO-LIA	WB reactivity	No.	qPCR results	No. of samples positive by the following assay/			
	reactivity pattern	pattern		(positive no./tested no.)	total no. of samples tested:			
								
					Avioq	Murex	Roche	Fujirebio
INNO-LIA±/WB−	gp21 I/II	/	127	0/30	4/130 (0.3%)	54/130 (41.53%)	93/130 (71.5%)	72/130 (55.38%)
	p19 I/II, p24 I/II	/	3	NT				
INNO-LIA−/WB±	/	rgp46-II	5	NT	2/15 (12.3%)	8/15 (53.3%)	9/15 (60.0%)	5/15 (33.3%)
	/	GD21	2					
	/	p19	3					
	p19 I/II	p19	1					
	p19 I/II	p24	1					
	p19 I/II	p19, p26	1					
	/	rgp46-I	1					
	p24 I/II	p24, rgp46-I	1					
INNO-LIA±/WB±	gp21 I/II	GD21	3	NT	0/6 (0.00%)	4/6 (66.67%)	4/6 (66.67%)	5/6 (83.3%)
	gp21 I/II	rgp46-I	1					
	gp21 I/II	rgp46-II	1					
	p19 I/II,p24 I/II	p19, p26	1					
Total			151	0/30	6/151 (0.4%)	66/151 (43.7%)	103/151 (68.7%)	82/151 (54.3%)

*LIA, line immunoassay; WB, western blot; ±, indeterminate; −, negative; NT, not tested.*

Thirteen indeterminate samples were recollected from the same donors no less than 8 weeks after last donation and retested by all assays (Supplementary Table S3). Seroconversion occurred in 2 individuals with LIA ± /WB- results. Both samples converted from the single gp21 I/II band pattern to the gp21 I/II plus p19 I/II band pattern but were still WB-negative. In addition, 4 out of the 13 indeterminate samples, including 2 LIA ± /WB- samples and 2 LIA-/WB ± samples, converted to negative. Six recollected samples were also retested by qPCR, but all of them were NAT-negative, including the seroconverted LIA-HTLV positive sample.

## Discussion

Currently, screening for HTLV-1/2 antibodies in healthy blood donors is not done routinely in China. A systematic review revealed that HTLV-1/2 prevalence in the coastal areas of China, and especially in Fujian province, is higher, compared to the low average HTLV-1/2 prevalence in the mainland (Chen et al., 2019). However, it is notable that in some provinces close to the high-endemic areas of China, including Hunan, Jiangsu and Guangxi, the prevalence is relatively high, probably due to large-scale population migration in these regions in recent years (Chen et al., 2019). Accordingly, HTLV-1/2 may have spread from the coastal areas to the mainland, resulting in increased opportunities for virus transmission and infection. Therefore, it is reasonable to expect HTLV-1/2 detection to become necessary in China. Consequently, appropriate strategies for screening and confirming HTLV-1/2 infection in China should be developed urgently.

In our study, almost one-third samples displayed discordant results among the four evaluated assays, which may due to the differences in the combination, source and quantity of HTLV-1/2 antigens used by different manufacturers. None of the evaluated assays could detect all the true HTLV positives when using LIA as a standard. In contrast, the Roche-ECLIA was a relatively more sensitive test that was reactive to 91.8% confirmed positive samples. This assay was especially sensitive to the untyped positives (7/12, 58.33%). Moreover, Roche-ECLIA could detect more indeterminate samples than the other three assays, indicating it was a sensitive method for screening HTLV-1/2 antibodies. From the perspective of blood banks/centers, sending RR samples to a reference lab is costly and time-consuming. Therefore, we propose that, where possible, such samples could be retested by the Roche-ECLIA and the positives subjected to subsequent confirmatory tests. Our data suggest that this would reduce the need for confirmation by about two thirds.

The present study indicated that most untypable HTLV infections had relatively low S/CO or COI values or were misdiagnosed by some screening tests, indicating a high risk of missed detections when only one screening assay is used in the reference labs. In our study, the combination of Roche-ECLIA and Murex-ELISA performed relatively well, detecting 98.4% true positives when LIA was the standard. We detected 100% positive samples when Murex-ELISA was combined with Roche-ECLIA and Fujirebio-CLIA but this strategy would be costly, time-consuming and labor intensive for reference labs.

HTLV-1-associated diseases develop in only a small proportion of infected individuals and slowly progress to disease relative to the average life span of humans. Consequently, after balancing screening/conformational costs and the threat of HTLV infections to public health especially in the non-endemic area, we recommend the use of a combination of Roche-ECLIA and Murex-ELISA in reference centers to retest initially reactive blood samples in low-risk areas. Since Roche-ECLIA is more sensitive than Murex-ELISA, it is best to test RR samples with Roche-ECLIA first and then the negatives by Murex-ELISA. Therefore, the number of test would be reduced by about 12% compared with test simultaneously.

Our results also indicate that higher S/CO or COI values were associated with higher diagnostic reliability. As demonstrated in this study, samples with S/CO values over 2.5, 11.0, 67.2 for Avioq-ELISA, Murex-ELISA, and Roche-ECLIA, respectively, or COI value over 28.0 for Fujirebio-CLIA, were rarely negative. Hence, samples with values above these thresholds can be interpreted as HTLV-1/2 positive, decreasing the demand and cost of confirmatory tests. Accordingly, if Murex-ELISA and Roche-ECLIA were combined for sample retesting in reference centers, the number of samples needing confirmation would reduce by 14.7%. Nevertheless, molecular assays or other supplementary tests would be necessary to determine PVL or HTLV infection types.

WB-indeterminate and/or untypable variants remain a serious problem worldwide. It has been found that the frequency of WB-indeterminate samples reactive to HTLV-1 ELSIA varies among different cohorts, but is much higher in low-risk blood donors (Martins et al., 2010; Abrams et al., 2011). In our study, only 4.5% (27/599) of WB-indeterminate samples and no WB untypable samples were identified. The low sensitivity of WB and the special specimens collected from blood donors in the HTLV-1 infected area may account for these results. Of the WB-indeterminate samples, 22.2% (6/27) were confirmed positive by LIA and 11.1% (3/27) were further confirmed HTLV-1 positive by qPCR. Although most of the WB-indeterminate, LIA-positive samples (4/6) were LIA untypable, the results indicated that LIA was more sensitive than WB and, in our view, should be the first choice for confirmation.

Indeterminate WB patterns indicating true or false virus infection have been reported previously (Martins et al., 2010). In our study, rgp46-II alone was the most frequently observed pattern, which was remarkably different from the pattern observed in blood donors or general population from other regions (Hayes et al., 1991; Mauclère et al., 1997; Lu and Chen, 2003; Filippone et al., 2012), indicating that indeterminate WB patterns can vary among populations. Martins et al. (2010) reported that the presence of p19 plus p24 seemed to indicate true reactivity and p24 alone indicated false reactivity. In our study, the p19 band was observed in 5/6 WB ± /LIA+ samples, 3 of which also exhibited another unique band (p24 or rgp46-I or GD21). Therefore, we suspected that the presence of p19 plus a unique GAG or ENV band indicated true HTLV infection and should be validated by LIA or molecular methods. In high-risk or endemic areas, indeterminate WB patterns may represent seroconverters (Jacob et al., 2008; Martins et al., 2010). In this study, seroindeterminate blood donors were usually recalled for recollection. However, only three WB-indeterminate samples were returned, two of which showed faint rgp46-I or rgp46-II bands and were reassigned as negative. We estimated that a proportion of the WB-indeterminate samples were false reactions, but larger follow-up studies to test this notion should be performed.

Although LIA reduced the number of WB-indeterminate samples, the assay yielded more indeterminate (22.7%, 136/599) and untypable (2.3%, 14/599) results than WB. Most of the samples expressed a single gp21 I/II pattern (97.1%, 132/136), the largest of which had faint intensity (55.1%, 75/136). We propose several reasons for these inconclusive results, including low HTLV proviral loads, mutations in the provirus (defective particles), seroconversion period or cross-reactivity with other antigens or viruses. In our study, 30 LIA-indeterminate and 5 LIA-untyped samples were tested by qPCR, but only one LIA-untyped sample was confirmed HTLV-1 positive with a PVL of 0.009 copies/100 PMBC, indicating that samples with very low PVL may be hard to identify. Manns et al. (1991) demonstrated that antibodies to rgp21 antigen appeared earlier than antibodies to p24 and p19, and the presence of the single rgp21 band may indicate an early HTLV infection. In our follow-up results, 2 out of 11 recollected samples with strong gp21 I/II intensity converted to HTLV-positive, suggesting a proportion of the LIA-indeterminate samples were in the seroconversion phase and needed to be followed-up. However, 2 samples with faint gp21 I/II intensity were reassigned as negative, revealing that some of the indeterminate samples may have resulted from non-specific reactivity or cross-reactivity with other antigens. Interestingly, samples with strong rgp21 I/II intensity displayed significantly higher S/CO or COI values than samples with faint intensity (Supplementary Figure S1). Taken together, the correlation between higher reactivity indexes and higher diagnostic reliability and the observation that the 2 LIA-indeterminate samples with strong rgp21 I/II intensity retested HTLV positive, support the view that LIA-indeterminate samples with strong intensities are more likely to still be in the seroconversion period, and should be paid serious attention.

Although the qPCR method used in our study did not detect all the specimens, our results indicated that qPCR was useful for resolving inconclusive confirmatory tests. Due to the large numbers of uncertain results and the high cost of WB and LIA assays, we propose employing qPCR first, followed by WB or LIA to test any qPCR-negatives. Other studies also recommend this strategy, which has been shown to reduce costs and improve the accuracy of HTLV-1/2 diagnosis (Costa et al., 2011; da Silva Brito et al., 2018).

Without a prophylactic vaccine and with limited available treatment options for HTLV-related diseases, recommendations for the deferral and reentry of blood donors are necessary, especially in China where HTLV-1/2 screening is underdeveloped. Based on our study, we suggest that donors with a final positive interpretation by a confirmatory test should be deferred permanently but donors returning a negative result should be reentered. Additionally, donors with indeterminate results should be followed-up, and recollected specimens should be confirmed through the same supplemental test.

## Conclusion

**FIGURE 3:**
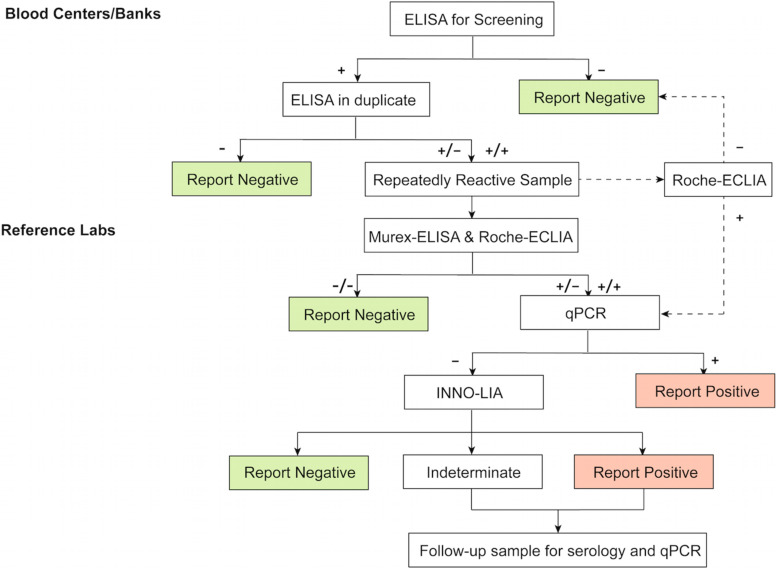
Test algorithm for screening and confirmation of HTLV-1/2 infection in low-endemic areas. ELISA, enzyme-linked immunosorbent assays, CLIA, chemiluminescence immunoassays; ECLIA, electrochemiluminescence immunoassays. +: positive/reactive; −: negative/non-reactive.

In this study, we compared the performance of four commercially available kits for screening anti-HTLV-1/2 antibodies and used WB and LIA as confirmatory tests. We also proposed a new testing algorithm for screening and confirming HTLV-1/2 in a low prevalence region (Figure 3). We recommend Roche-ECLIA as a second screening test in blood centers/banks to rule out large numbers of false-positives. Where local equipment is lacking, all RR specimens would need to be confirmed in reference labs. To minimize the risk of misdiagnosing true positives, samples that are reactive to Murex-ELISA or Roche-ECLIA should be further confirmed. Taking the cost into consideration, we suggest that these should be quantified using qPCR first, and then the negatives should be subjected to LIA but not WB to improve the diagnostic accuracy.

## Data Availability Statement

The original contributions presented in the study are included in the article/Supplementary Material, further inquiries can be directed to the corresponding authors.

## Ethics Statement

This study was approved by the institutional review boards in every participating blood bank. Written informed consent are routinely completed at the time of donation from blood donors.

## Author Contributions

HJ, LC, andLWdesigned the study and wrote the manuscript. HJ, LC, XJ, HS, and FG performed the experiments. HJ, LC, YY, and LW analyzed the data. All authors participated in critical revision of the manuscript and approved the final version.

## Conflict of Interest

The authors declare that the research was conducted in the absence of any commercial or financial relationships that could be construed as a potential conflict of interest.

## Abbreviations

CLIA: chemiluminescence immunoassay
ECLIA: electrochemiluminescence immunoassay
ELISA: enzyme-linked immunosorbent assay
HTLV-1/2: human T-cell lymphotropic virus type ½
LIA: line immunoassay
NCCL: National Center for Clinical Laboratories
PPV: positive predictive value
RR: repeatedly reactive
WB: western blot.

## References

[B1] Abrams A. Akahata Y. Jacobson S. (2011). The prevalence and significance of HTLV-I/II seroindeterminate Western blot patterns. *Viruses* 3 1320–1331. 10.3390/v3081320 21994781 PMC3185804

[B2] Andersson S. Thorstensson R. Ramirez K. G. Krook A. Von Sydow M. Dias F. (1999). Comparative evaluation of 14 immunoassays for detection of antibodies to the human T-lymphotropic virus types I and II using panels of sera from Sweden and West Africa. *Transfusion* 39 845–851. 10.1046/j.1537-2995.1999.39080845.x 10504120

[B3] Ando Y. Ekuni Y. Matsumoto Y. Nakano S. Saito K. Kakimoto K. (2004). Long-term serological outcome of infants who received frozen-thawed milk from human T-lymphotropic virus type-I positive mothers. *J. Obstetr. Gynaecol. Res.* 30 436–438. 10.1111/j.1447-0756.2004.00227.x 15566458

[B4] Calattini S. Chevalier S. A. Duprez R. Bassot S. Froment A. Mahieux R. (2005). Discovery of a new human T-cell lymphotropic virus (HTLV-3) in Central Africa. *Retrovirology* 2:30. 10.1186/1742-4690-2-30 15882466 PMC1142341

[B5] Campos K. R. Gonçalves M. G. Caterino-de-Araujo A. (2017). Short communication: failures in detecting HTLV-1 and HTLV-2 in patients infected with HIV-1. *AIDS Res. Hum. Retrovirus.* 33 382–385. 10.1089/AID.2016.0191 27750018

[B6] Chen X. Liu F. Fu X. Feng Y. Zhang D. Liu H. (2019). Prevalence of human T-cell lymphotropic virus type-1 infection among blood donors in mainland China: a systematic review and meta-analysis of the last 20 years. *Expert Rev. Hematol.* 12 579–587. 10.1080/17474086.2019.1632703 31220418

[B7] Costa E. A. S. Magri M. C. Caterino-de-Araujo A. (2011). The best algorithm to confirm the diagnosis of HTLV-1 and HTLV-2 in at-risk individuals from São Paulo, Brazil. *J. Virol. Methods* 173 280–286. 10.1016/j.jviromet.2011.02.018 21349293

[B8] da Silva Brito V. Santos F. L. N. Gonçalves N. L. S. Araujo T. H. A. Nascimento D. S. V. Pereira F. M. (2018). Performance of commercially available serological screening tests for human T-cell lymphotropic virus infection in Brazil. *J. Clin. Microbiol.* 56 8–14. 10.1128/JCM.00961-18 30232131 PMC6258847

[B9] Filippone C. Bassot S. Betsem E. Tortevoye P. Guillotte M. Mercereau-Puijalon O. (2012). a new and frequent human T-Cell leukemia virus indeterminate western blot pattern: epidemiological determinants and PCR results in central African inhabitants. *J. Clin. Microbiol.* 50 1663–1672. 10.1128/JCM.06540-11 22403426 PMC3347141

[B10] Hayes C. G. Burans J. R. Oberst R. B. (1991). Antibodies to human t lymphotropic virus type i in a population from the philippines: evidence for cross-reactivity with plasmodium falciparum. *J. Infect. Dis.* 163 257–262. 10.1093/infdis/163.2.257 1988510

[B11] Jacob F. Santos-Fortuna E. Azevedo R. S. Caterino-de-Araujo A. (2008). Serological patterns and temporal trends of HTLV-1/2 infection in high-risk populations attending Public Health Units in São Paulo, Brazil. *J. Clin. Virol.* 42 149–155. 10.1016/j.jcv.2008.01.017 18346935

[B12] Kalyanaraman V. S. Sarngadharan M. G. Robert-Guroff M. Miyoshi I. Blayney D. Golde D. (1982). A new subtype of human T-cell leukemia virus (HTLV-II) associated with a T-cell variant of hairy cell leukemia. *Science* 218 571–573. 10.1126/science.6981847 6981847

[B13] Lu S. C. Chen B. H. (2003). Seroindeterminate HTLV-1 prevalence and characteristics in blood donors in Taiwan. *Int. J. Hematol.* 77 412–413. 10.1007/BF02982654 12774934

[B14] Maeda Y. (1989). Prevention of transmission of human T-lymphotropic virus type 1 (HTLV-1) through transfusion, by donor screening with antibody to the virus One-year experience. *Transfusion* 29 7–11. 10.1046/j.1537-2995.1989.29189101168.x 2643213

[B15] Mahieux R. Horal P. Mauclère P. Mercereau-Puijalon O. Guillotte M. Meertens L. (2000). Human T-cell lymphotropic virus type 1 Gag indeterminate western blot patterns in Central Africa: relationship to *Plasmodium falciparum* infection. *J. Clin. Microbiol.* 38 4049–4057. 10.1128/jcm.38.11.4049-4057.2000 11060067 PMC87540

[B16] Manns A. Murphy E. L. Wilks R. Haynes G. Figueroa J. P. Hanchard B. (1991). Detection of early human T-cell lymphotropic virus type I antibody patterns during seroconversion among transfusion recipients. *Blood* 77 896–905. 10.1182/blood.v77.4.896.bloodjournal774896 1993227

[B17] Martins M. L. Da Silva Santos A. C. Namen-Lopes M. S. Barbosa-Stancioli E. F. Utsch D. G. De Carneiro-Proietti A. B. F. (2010). Long-term serological follow-up of blood donors with an HTLV-indeterminate western blot: antibody profile of seroconverters and individuals with false reactions. *J. Med. Virol.* 82 1746–1753. 10.1002/jmv.21881 20827773

[B18] Mauclère P. Le Hesran J. Mahieux R. Salla R. Mfoupouendoun J. Abada E. T. (1997). Demographic, ethnic, and geographic differences between human T Cell lymphotropic virus (HTLV) Type I-*Seropositive Carriers* and Persons with HTLV-I gag-indeterminate Western blots in central Africa. *J. Infect. Dis.* 176 505–540. 10.1086/514071 9237719

[B19] Montanheiro P. Olah I. Fukumori L. M. I. Smid J. Oliveira A. C. P. de Kanzaki L. I. B. (2008). Low DNA HTLV-2 proviral load among women in São Paulo City. *Virus Res.* 135 22–25. 10.1016/j.virusres.2008.01.015 18343520

[B20] Poiesz B. J. Ruscetti F. W. Gazdar A. F. Bunn P. A. Minna J. D. Gallo R. C. (1980). Detection and isolation of type C retrovirus particles from fresh and cultured lymphocytes of a patient with cutaneous T-cell lymphoma. *Proc. Natl. Acad. Sci. U.S.A.* 77 7415–7419. 10.1073/pnas.77.12.7415 6261256 PMC350514

[B21] Varma M. Rudolph D. L. Knuchel M. Switzer W. M. Hadlock K. G. Velligan M. (1995). Enhanced specificity of truncated transmembrane protein for serologic confirmation of human T-Cell lymphotropic virus type 1 (HTLV-1) and HTLV-2 infections by Western blot (immunoblot) assay containing recombinant envelope glycoproteins. *J. Clin. Microbiol.* 33 3239–3244. 10.1128/jcm.33.12.3239-3244.1995 8586709 PMC228680

[B22] Verdonck K. González E. Van Dooren S. Vandamme A. M. Vanham G. Gotuzzo E. (2007). Human T-lymphotropic virus 1: recent knowledge about an ancient infection. *Lancet Infect. Dis.* 7 266–281. 10.1016/S1473-3099(07)70081-6 17376384

[B23] Williams A. E. Fang C. T. Slamon D. J. Poiesz B. J. Sandler S. G. Darr W. F. (1988). Seroprevalence and epidemiological correlates of HTLV-I infection in U.S. blood donors. *Science* 240 643–646. 10.1126/science.2896386 2896386

[B24] Wolfe N. D. Heneine W. Carr J. K. Garcia A. D. Shanmugam V. Tamoufe U. (2005). Emergence of unique primate T-lymphotropic viruses among central African bushmeat hunters. *Proc. Natl. Acad. Sci. U.S.A.* 102 7994–7999. 10.1073/pnas.0501734102 15911757 PMC1142377

[B25] Yao K. Hisada M. Maloney E. Yamano Y. Hanchard B. Wilks R. (2006). Human T lymphotropic virus types I and II Western blot seroindeterminate status and its association with exposure to prototype HTLV-I. *J. Infect. Dis.* 193 427–437. 10.1086/499273 16388491

[B26] Zrein M. Louwagie J. Boeykens H. Govers L. Hendrickx G. Bosman F. (1998). Assessment of a new immunoassay for serological confirmation and discrimination of human T-cell lymphotropic virus infections. *Clin. Diagn. Lab. Immunol* 5 45–49. 10.1128/cdli.5.1.45-49.1998 9455879 PMC121390

