# Supplementary material for: A Strategy for Screening and Confirmation of HTLV-1/2 Infections in Low-Endemic Areas
Source: Front Microbiol. 2020 Jun 3;11:1151. doi: 10.3389/fmicb.2020.01151 (PMC7283491; doi:10.3389/fmicb.2020.01151)
Supplement: Supplementary file 1 [file Data_Sheet_1.docx]

Supplementary Materials for

**Strategy for screening and confirmation of HTLV-1/2 infection in low-endemic areas**

Huimin Ji ^123^, Le Chang ^12^, Ying Yan^12^, Xinyi Jiang ^123^, Huizhen Sun^123^, Fei Guo^12^, Lunan Wang^123 ＃^

1. National Center for Clinical Laboratories, Beijing Hospital, National Center of Gerontology; Institute of Geriatric Medicine, Chinese Academy of Medical Sciences, Beijing, P. R. China

2. Beijing Engineering Research Center of Laboratory Medicine, Beijing Hospital, Beijing, P. R. China

3. Graduate School, Peking Union Medical College, Chinese Academy of Medical Sciences, Beijing, P. R. China

^＃^Corresponding Author:

Lunan Wang, Email: [lunan99@163.com](mailto:lunan99@163.com)

Tel.: + 86 10 85133609


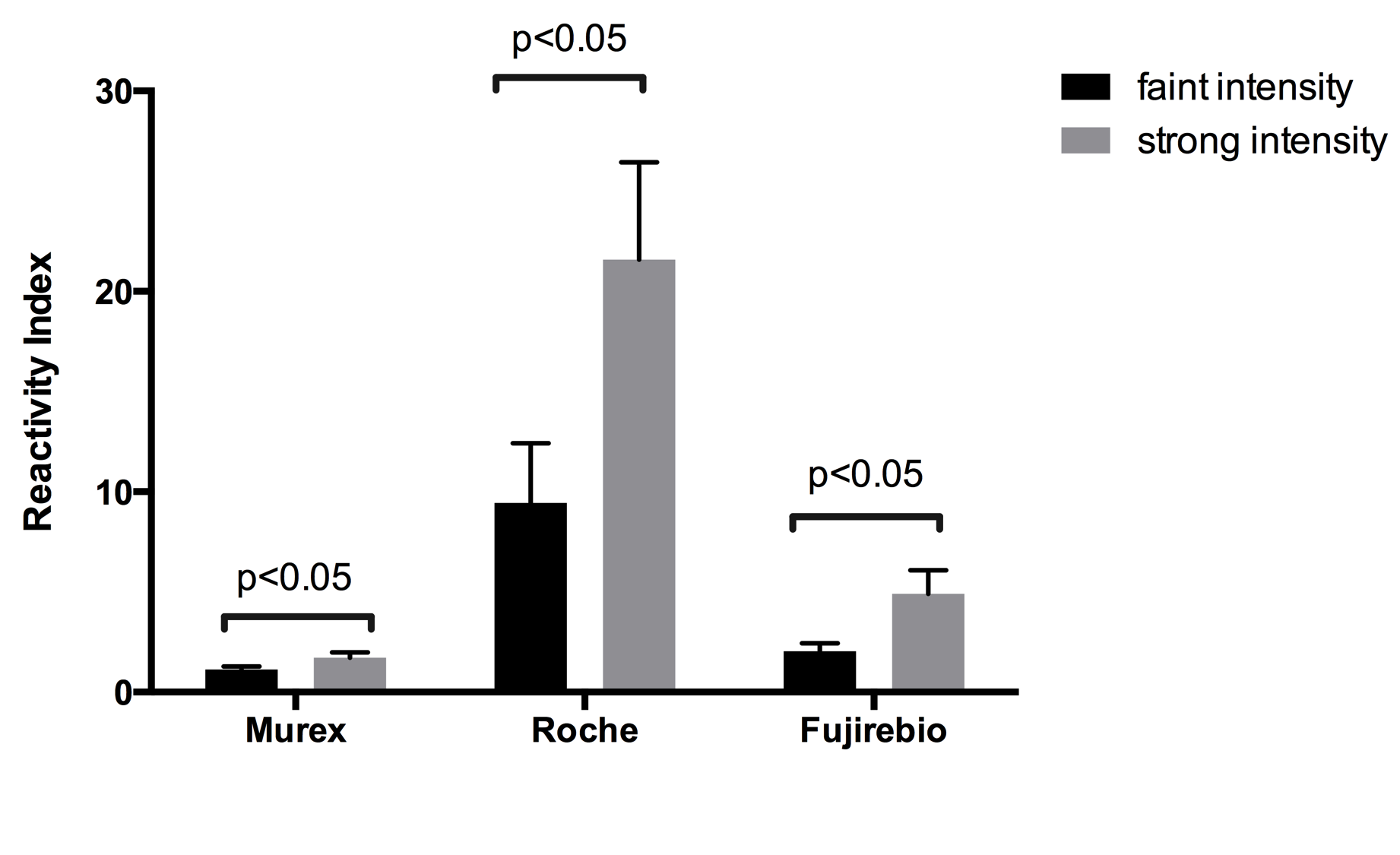


Fig. S1. Comparison of S/CO or COI values between the indeterminate samples with faint and strong rgp21 I/II intensity.

Note: faint intensity: the rgp21 I/II intensity equals to “±” when compared to the control line; strong intensity: the rgp21 I/II intensity ranging from 1+ to 3+ when compared to the control line. Data shown are mean ± SD.

Table S1. Primers and probes used in this study

| Names | 5' - 3' Sequences |
| --- | --- |
| HTLV-1-Forward | CTAGCCCACTTGCAAACTATAGACCT |
| HTLV-1-Reverse | CGTAGTTACACTGCTGTGGGACAG |
| HTLV-1-probe | AGACGCCTTTTTCCAAATCCCCTTACC |
| RPPH1- Forward | CATGCCGTCCCGCGATATTGA |
| RPPH1- Reverse | GTTGATGACGTCAGCGTTCGAATTC |
| RPPH1-probe | CTCCGAACCTCTCGCCCTGCC |

**Table S2. Details of the consistant results by LIA and WB among the finally confirmed HTLV-1 positive samples**

| Sample No. |  | ELISA | |  | CLIA | |  | INNO-LIA |  | WB |  | qPCR |
| --- | --- | --- | --- | --- | --- | --- | --- | --- | --- | --- | --- | --- |
|  |  | Avioq | Murex |  | Roche | Fujirebio |  | resutls |  | results |  | Result |
|  |  | S/CO | S/CO |  | S/CO | COI |  |  |  |  |  |  |
| 4 |  | 7.516 | 11.212 |  | 329.3 | 50.0 |  | HTLV-1 |  | HTLV-1 |  | NT |
| 7 |  | 7.605 | 11.135 |  | 469.1 | 50.0 |  | HTLV-1 |  | HTLV-1 |  | NT |
| 36 |  | 4.782 | 9.814 |  | 139 | 50.0 |  | HTLV-1 |  | HTLV-1 |  | NT |
| 88 |  | 3.960 | 9.728 |  | 447.7 | 50.0 |  | HTLV-1 |  | HTLV-1 |  | NT |
| 109 |  | 4.632 | 9.764 |  | 358.3 | 50.0 |  | HTLV-1 |  | HTLV-1 |  | NT |
| 146 |  | 6.531 | 8.963 |  | 310.2 | 50.0 |  | HTLV-1 |  | HTLV-1 |  | NT |
| 148 |  | 3.186 | 9.003 |  | 238.8 | 50.0 |  | HTLV-1 |  | HTLV-1 |  | NT |
| 153 |  | 5.131 | 9.477 |  | 314.3 | 50.0 |  | HTLV-1 |  | HTLV-1 |  | NT |
| 163 |  | 5.869 | 9.509 |  | 435.7 | 50.0 |  | HTLV-1 |  | HTLV-1 |  | NT |
| 188 |  | 6.025 | 9.180 |  | 481.7 | 50.0 |  | HTLV-1 |  | HTLV-1 |  | NT |
| 189 |  | 6.098 | 12.997 |  | 488.2 | 50.0 |  | HTLV-1 |  | HTLV-1 |  | NT |
| 220 |  | 1.763 | 9.935 |  | 276.5 | 50.0 |  | HTLV-1 |  | HTLV-1 |  | NT |
| 409 |  | 3.030 | 5.783 |  | 264.3 | 50.0 |  | HTLV-1 |  | HTLV-1 |  | NT |
| 434 |  | 5.478 | 9.051 |  | 402.1 | 50.0 |  | HTLV-1 |  | HTLV-1 |  | NT |
| 540 |  | 9.30 | 12.54 |  | 420.3 | 50.0 |  | HTLV-1 |  | HTLV-1 |  | NT |
| 605 |  | 3.37 | 11.06 |  | 380.2 | 50.0 |  | HTLV-1 |  | HTLV-1 |  | NT |
| 649 |  | 3.74 | 10.42 |  | 271.3 | 50.0 |  | HTLV-1 |  | HTLV-1 |  | NT |
| 820 |  | 6.03 | 10.09 |  | 452.1 | 50.0 |  | HTLV-1 |  | HTLV-1 |  | NT |
| 907 |  | 2.43 | 10.08 |  | 410.2 | 50.0 |  | HTLV-1 |  | HTLV-1 |  | NT |
| 918 |  | 4.43 | 12.61 |  | 262.1 | 50.0 |  | HTLV-1 |  | HTLV-1 |  | NT |
| 956 |  | 5.53 | 6.12 |  | 83.79 | 28.4 |  | HTLV-1 |  | HTLV-1 |  | NT |
| 960 |  | 5.66 | 8.84 |  | 358.9 | 50.0 |  | HTLV-1 |  | HTLV-1 |  | NT |
| 1081 |  | 6.28 | 10.89 |  | 251.8 | 50.0 |  | HTLV-1 |  | HTLV-1 |  | NT |
| 1113 |  | 6.13 | 10.65 |  | 355.3 | 50.0 |  | HTLV-1 |  | HTLV-1 |  | NT |
| 1120 |  | 5.81 | 10.86 |  | 423.7 | 50.0 |  | HTLV-1 |  | HTLV-1 |  | NT |
| 1124 |  | 6.86 | 10.44 |  | 504.7 | 50.0 |  | HTLV-1 |  | HTLV-1 |  | NT |
| 1169 |  | 5.71 | 12.13 |  | 509.7 | 50.0 |  | HTLV-1 |  | HTLV-1 |  | NT |
| 1197 |  | 6.94 | 11.18 |  | 353.2 | 50.0 |  | HTLV-1 |  | HTLV-1 |  | NT |
| 1198 |  | 19.73 | 13.11 |  | 361 | 50.0 |  | HTLV-1 |  | HTLV-1 |  | NT |
| 1381 |  | 5.88 | 12.65 |  | 449 | 50.0 |  | HTLV-1 |  | HTLV-1 |  | NT |
| 1387 |  | 5.78 | 12.71 |  | 521.4 | 50.0 |  | HTLV-1 |  | HTLV-1 |  | NT |
| 1405 |  | 4.02 | 12.41 |  | 66.54 | 50.0 |  | HTLV-1 |  | HTLV-1 |  | NT |
| 1407 |  | 6.18 | 13.43 |  | 274.6 | 50.0 |  | HTLV-1 |  | HTLV-1 |  | NT |
| 1409 |  | 6.67 | 13.22 |  | 376 | 50.0 |  | HTLV-1 |  | HTLV-1 |  | NT |
| 1488 |  | 7.23 | 12.46 |  | 514.1 | 50.0 |  | HTLV-1 |  | HTLV-1 |  | NT |
| 1594 |  | 2.561 | 3.429 |  | 402 | 50.0 |  | HTLV-1 |  | HTLV-1 |  | NT |
| 1598 |  | 2.108 | 3.864 |  | 361.3 | 50.0 |  | HTLV-1 |  | HTLV-1 |  | NT |
| 1312 |  | 7.39 | 11.06 |  | 450.2 | 50.0 |  | HTLV-1 |  | HTLV-1 |  | Pos |
| 1648 |  | 1.821 | 3.389 |  | 365.2 | 50.0 |  | HTLV-1 |  | HTLV-1 |  | Pos |
| 1757 |  | 1.719 | 3.507 |  | 364.6 | 50.0 |  | HTLV-1 |  | HTLV-1 |  | Pos |
| 94 |  | 0.056 | 10.759 |  | 87.7 | 50.0 |  | HTLV-1 |  | HTLV-1 |  | NT |
| 186 |  | 0.312 | 8.987 |  | 283.7 | 50.0 |  | HTLV-1 |  | HTLV-1 |  | NT |
| 1607 |  | 0.066 | 3.338 |  | 512.9 | 50.0 |  | HTLV-1 |  | HTLV-1 |  | Pos |
| 1082 |  | 0.88 | 0.35 |  | 23.49 | 43.0 |  | HTLV-1 |  | HTLV-1 |  | NT |

Table S3. Results and band petterns of WB 2.4 and LIA of 13 recollected indeterminate specimens

| Sample No. | ELISA | |  | CLIA | |  | INNO-LIA | |  | WB 2.4 | |  | qPCR |
| --- | --- | --- | --- | --- | --- | --- | --- | --- | --- | --- | --- | --- | --- |
|  | Avioq | Murex |  | Roche | Fujirebio |  | Bands Pattern (band intensity) | Results |  | Bands Pattern | Results |  | Results |
|  | S/CO | S/CO |  | S/CO | COI |  |  |  |  |  |  |  |  |
| 91 | 0.148 | 0.564 |  | 42.37 | 0.5 |  | gp21 I/II（1+） | Ind |  | / | Neg |  | NT |
|  | 0.172 | 0.895 |  | 59.43 | 0.4 |  | gp21 I/II（±） | Ind |  | / | Neg |  | NT |
| 177 | 0.426 | 1.538 |  | 3.71 | 6.1 |  | gp21 I/II（1+） | Ind |  | / | Neg |  | NT |
|  | 0.484 | 0.540 |  | 4.11 | 9.5 |  | p19 I/II (1+), gp21 I/II（±） | HTLV |  | / | Neg |  | NT |
| 513 | 0.25 | 1.13 |  | 34.48 | 50 |  | gp21 I/II（2+） | Ind |  | p24，rgp46-I | Ind |  | NT |
|  | 0.23 | 1.04 |  | 66.61 | 49.1 |  | gp21 I/II（±） | Ind |  | p24，rgp46-I | Ind |  | NT |
| 1342 | 0.65 | 4.89 |  | 171.5 | 19.4 |  | gp21 I/II（2+） | Ind |  | / | Neg |  | Neg |
|  | 1.11 | 5.51 |  | 142.1 | NT |  | p19 I/II (±), gp21 I/II（2+） | HTLV |  | / | Neg |  | Neg |
| 1357 | 0.17 | 0.68 |  | 197.1 | 0.2 |  | gp21 I/II（±） | Ind |  | / | Neg |  | Neg |
|  | 0.12 | 0.38 |  | 213.2 | NT |  | gp21 I/II（±） | Ind |  | / | Neg |  | Neg |
| 1439 | 0.21 | 2.05 |  | 27.3 | 1.7 |  | gp21 I/II（±） | Ind |  | / | Neg |  | Neg |
|  | 0.13 | 0.35 |  | 0.201 | 1.3 |  | gp21 I/II（±） | Ind |  | / | Neg |  | NT |
| 1442 | 0.35 | 2.85 |  | 0.607 | 1.1 |  | gp21 I/II（±） | Ind |  | / | Neg |  | Neg |
|  | 0.19 | 2.91 |  | 0.599 | 0.2 |  | ／ | Neg |  | / | Neg |  | NT |
| 1454 | 0.42 | 0.46 |  | 8.05 | 5.1 |  | gp21 I/II（2+） | Ind |  | / | Neg |  | Neg |
|  | 0.035 | 0.104 |  | NT | NT |  | gp21 I/II（2+） | Ind |  | / | Neg |  | Neg |
| 1627 | 0.088 | 0.245 |  | 2.3 | 0.4 |  | gp21 I/II（±） | Ind |  | / | Neg |  | Neg |
|  | 0.067 | 0.129 |  | 1.9 | 0.4 |  | ／ | Neg |  | / | Neg |  | Neg |
| 1693 | 0.054 | 2.458 |  | 3.62 | 5.8 |  | gp21 I/II（1+） | Ind |  | / | Neg |  | Neg |
|  | 0.021 | 1.928 |  | NT | NT |  | gp21 I/II（2+） | Ind |  | / | Neg |  | Neg |
| 1733 | 0.063 | 1.95 |  | 0.485 | 2.6 |  | gp21 I/II（±） | Ind |  | / | Neg |  | Neg |
|  | 0.033 | 1.99 |  | NT | NT |  | ／ | Neg |  | / | Neg |  | Neg |
| 252 | 0.173 | 0.663 |  | 7.97 | 0.1 |  | ／ | Neg |  | rgp46-I | Ind |  | NT |
|  | 0.294 | 0.444 |  | NT | NT |  | ／ | Neg |  | / | Neg |  | NT |
| 429 | 0.258 | 0.437 |  | 1.54 | 0.1 |  | ／ | Neg |  | rgp46-II | Ind |  | NT |
|  | 0.191 | 0.373 |  | NT | NT |  | ／ | Neg |  | / | Neg |  | NT |

Note: LIA, line immunoassay; WB, Western Blot; PVL, proviral load; Ind, indeterminate, Pos, Positive; Neg, Negative; NT, Not tested.
